# Natural Selection Plays an Important Role in Shaping the Codon Usage of Structural Genes of the Viruses Belonging to the *Coronaviridae* Family

**DOI:** 10.3390/v13010003

**Published:** 2020-12-22

**Authors:** Dimpal A. Nyayanit, Pragya D. Yadav, Rutuja Kharde, Sarah Cherian

**Affiliations:** 1 Maximum Containment Facility, ICMR-National Institute of Virology, Sus Road, Pashan, Pune 411021, India; nyayanit.dimpal@gmail.com (D.A.N.); hellopragya22@gmail.com (P.D.Y.); rutujakharde13@gmail.com (R.K.); 2 Bioinformatics Group, ICMR-National Institute of Virology, Pune 411001, India

**Keywords:** codon usage bias, *Coronaviridae*, mutational selection, natural selection

## Abstract

Viruses belonging to the *Coronaviridae* family have a single-stranded positive-sense RNA with a poly-A tail. The genome has a length of ~29.9 kbps, which encodes for genes that are essential for cell survival and replication. Different evolutionary constraints constantly influence the codon usage bias (CUB) of different genes. A virus optimizes its codon usage to fit the host environment on which it savors. This study is a comprehensive analysis of the CUB for the different genes encoded by viruses of the *Coronaviridae* family. Different methods including relative synonymous codon usage (RSCU), an Effective number of codons (ENc), parity plot 2, and Neutrality plot, were adopted to analyze the factors responsible for the genetic evolution of the *Coronaviridae* family. Base composition and RSCU analyses demonstrated the presence of A-ended and U-ended codons being preferred in the 3rd codon position and are suggestive of mutational selection. The lesser ENc value for the spike ‘*S*’ gene suggests a higher bias in the codon usage of this gene compared to the other structural genes. Parity plot 2 and neutrality plot analyses demonstrate the role and the extent of mutational and natural selection towards the codon usage pattern. It was observed that the structural genes of the *Coronaviridae* family analyzed in this study were at the least under 84% influence of natural selection, implying a major role of natural selection in shaping the codon usage.

## 1. Introduction

The *Coronaviridae* family has four genera *Alphacoronaviridae*, *Betacoronaviridae*, *Gammacoronaviridae*, and *Deltacoronaviridae* that include 23 subgenera [1]. *Coronaviridae* families have single-stranded positive-sense RNA with a genome range of 26–32 kb in length, which is capped and polyadenylated [1]. The genome of the virus encodes for structural, non-structural, and accessory proteins. The four structural proteins encoded by the genome are envelope protein (E), membrane protein (M), nucleocapsid protein (N), and spike glycoprotein (S). *ORF1ab* gene encodes for pp1ab polyprotein and pp1a polyprotein, which are further spliced to obtain 15 different proteins [2].

The nucleocapsid proteinplays an important role in maintaining the RNA conformation stable for the replication, transcription, and translation of the viral genome along with protecting the viral genome [1,3]. It is highly immunogenic and capable of modulating the metabolism of an infected cell [3]. The envelope protein acts as a viroporin [4,5] and plays multiple roles in viral replication [1] and signaling pathways that affect inflammatory and type 1 INF gamma signaling [6]. The spike protein “S” is responsible for receptor recognition and membrane fusion [7] that leads to viral entry into the host cells [8]. The membrane protein is associated with the spike protein and is responsible for the virus budding process [9].

Different factors influence the gene translation process, a mechanism by which a protein is encoded. External factors (mutational pressure and natural selection process), along with internal factors (translational machinery of the organism), influences the gene translation, leading to the uneven usage of codons [10]. Genetic code redundancy leads to the biased usage of the codons under the influence of different factors, as observed in most of the organisms [11,12,13]. Genetic code redundancy is a term used when a single amino acid can be encoded by different codons. Codon usage bias (CUB) is caused due to the redundancy of the genetic code. As a result, each organism favors a particular set of synonymous codons. The result of this is that an individual codon is either used optimally or has a rare usage. Evolution shapes codon bias, and exploring this bias may facilitate our understanding of the viral genome evolution. A single genome with different genes may have different CUB or can have the same codon usage bias [14]. Recently differential choice of codon bias was proved by Deka et al. for matrix 1 and matrix 2 proteins encoded for Influenza A virus [15].

Each gene in the *Coronaviridae* family plays an essential role in the viral replication survival and infection, due to which it becomes essential to analyze the evolutionary factors involved in determining its choice for the codon bias. A recent paper by Tort et al. suggested mutational pressure to be the major factor responsible for shaping the codon usage pattern (CUP) in the severe acute respiratory syndrome coronavirus 2 (SARS-CoV-2) [16]. In another study by Anwar et al., natural selection and other factors were considered to shape the CUP of the SARS-CoV-2 [17]. A study by Dulicca et al. demonstrated the influence of both mutational and selectional pressures in shaping the CUP of the SARS-CoV-2 genes [18]. In this study, we analyzed the synonymous CUP of representative *Coronaviridae* sequences available in the GenBank database. It was observed that the nucleotide composition influences the codon usage of the different genes to different extents. Further, we studied the role of mutational pressure and natural selection on the evolution of the codon usage of the different genes, as observed in the *Coronaviridae* family.

## 2. Materials and Methods

Complete genome sequences of the viruses from the *Coronaviridae* family were obtained from the GenBank database of the National Center for Biotechnology Information (NCBI, USA). The list of the names of the viruses with their accession number is provided in Supplementary Table S1. Similar genes from different viruses were grouped into a single set. Likewise, different gene sets were obtained each for *N, M, S, E* genes.

### 2.1. Nucleotide Compositional Analysis

Nucleotide composition for the different gene sets was obtained using MEGA software v. 7.0 [19]. Further, the nucleotide compositional analysis was carried out for the various sets of genes belonging to *Coronaviridae* in terms of (i) overall percentage of each nucleotide in the gene (A%, G%, C%, and T%), (ii) G + C at the first, second and third position of the codon for each gene (GC1, GC2, and GC3) and (iii) overall AU and GC percentage in each gene. Factor analysis was carried out in “R”software to understand the overall nucleotide distribution for the different gene sets of *Coronaviridae.*

### 2.2. Relative Synonymous Codon Usage (RSCU) Analysis

Relative Synonymous Codon Usage (RSCU) is the fraction of the observed codon frequency to expected codon frequency, given that all the codons for any particular amino acid are used equally [20]. RSCU value for each gene is calculated using the Equation (1), as previously described by Sharp and Li [20].

(1) $$RSCU=\frac{f_{ij}}{\sum_{j}^{k_{i}}f_{ij}}k_{i}$$

where f_ij_ is the observed number of the i^th^ codon for the j^th^ amino acid, which has k_i_ kinds of synonymous codons. Three different types of RSCU values are obtained: (i) codon with RSCU < 1 are less frequently used and have a negative usage bias, (ii)codon with RSCU > 1 are frequently used and have positive usage bias and (iii) codon with RSCU = 1 has no bias. The RSCU values for the different gene sets were calculated using CodonW software v.1.4.2 and used for further analysis. For the RSCU values < 0.6, the codon is considered as under-represented and >1.6; it is considered as over-represented [21,22,23].

### 2.3. Role of Mutational Pressure on Codon Selection

#### The Effective Number of Codon (ENc) Analysis

The extent of variation in the codon usage bias for a gene can be determined using ENc. ENc value ranges from 20 to 61 [24]. An ENc value of 20 depicts an extreme CUB (using a single possible synonymous codon), whereas an ENc value of 61 is indicative of no bias (using all possible synonymous codons equally). ENc value is calculated using the Equation (2)

(2) $$ENc=2+\frac{9}{\bar{F_{2}}}+\frac{1}{\bar{F_{3}}}+\frac{5}{\bar{F_{4}}}+\frac{3}{\bar{F_{6}}}$$

where F_k_(k = 2, 3, 4, 6) is the mean of F_k_ values for *k*-fold degenerate amino acids. The values for F_k_ can be calculated using Equation (3) given below (Wright, 1990).

(3) $$\bar{F_{k}}=\frac{n\sum_{j=1}^{k}{\left(\frac{n_{j}}{n}\right)}^{2}-1}{n-1}$$

where “n” is the total occurrence of the codons for that amino acid and *n_j_* is the total occurrence of the j^th^ codon for that amino acid. The CodonW software was used to obtain the ENc values of all the gene sets.

The ENc values obtained are plotted against the GC3 values to measure the factor responsible for bias in codon usage. The expected ENc value is calculated using Equation (4)

(4) $$ENc_{\exp ected}=2+a+\frac{29}{a^{2}+(1-a^{2})}$$

where “*a”* denotes GC3s value. If the predicted ENc value has a standard deviation on the higher side than the standard curve of expected ENc value, then natural selection plays a significant role in the codon bias of the gene. If the predicted Enc value lies on or has a standard deviation less compared to the expected ENc value, then the codon bias of the gene is controlled by mutational pressure [25].

### 2.4. Role of Natural Selection on Codon Selection

#### 2.4.1. Parity Rule 2 Analysis

The Parity rule 2 (PR-2) analysis was performed to determine the role of selection and mutational pressure on the codon usage of different genes. PR-2 is the plot for the purine and pyrimidine usage at the third position of the four-fold degenerate amino acids. The value at the center position of both the axes determines the unbiased usage of the codons.

#### 2.4.2. Neutrality Plot

A neutrality plot was used to determine the extent of natural selection and mutational pressure influence on the codon usage bias of a gene. The neutrality plot was drawn using the average GC at the first and second position of the codon (GC12) versus GC at the third position of the codon GC3 values for each gene [26]. The distance between the group and within the group is calculated using MEGA software.

## 3. Results

### 3.1. Nucleotide Compositional Analysis

The nucleotide analysis demonstrates an overall highest mean for Thymine/Uracil (T/U), whereas Cytosine (C) has the lowest mean percentage of occurrence in all the genes sets analyzed (Table 1A). The mean percentage occurrence of U was highest (37.7 ± 3.5) in the *E* gene and lowest (23.1 ± 2.4) in the *N* gene (Table 1A). The mean highest percentage of occurrence for the C, A and G was 23.9 ± 2.3, 29.8 ± 1.9 and 23.0 ± 1.7 for the *N* gene. The lowest percentage of occurrence for the C, A and G was observed for *ORF1a* (17.5 ± 2.5), *M* (25.3 ± 1.8) and *ORF8* (18.2 ± 1.2). Overall, the observation of individual nucleotide frequencies indicates the preference for A and U nucleotides. 

Table 1B depicts the mean percentages for the sum of AU and GC nucleotides along with their standard deviation at the first, second and third positions. It is observed that either A or U are the preferred nucleotides at all three codon positions (Table 1B). The nucleotides A or U are most preferred at the third codon position. The mean value for the sum of AU leads to the overall observation that the virus belonging to the *Coronaviridae* family has an overall AU rich genome. A significant difference is observed in the GC content and the AU content at the second and third wobbles positions for all the genes.

The means of nucleotides, as well as the sum of dinucleotide at each codon position, indicate compositional codon bias in the genes of the *Coronaviridae* family (Table 1).

### 3.2. RSCU Analysis

**Figure 1:**
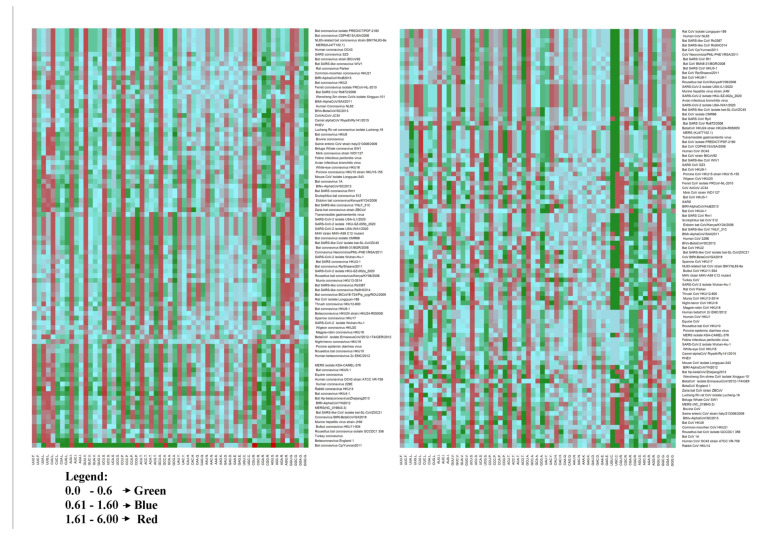
Heat map of the *S* and *N* genes based on relative synonymous codon usage (RSCU) values: A heat map plot of *S* (Left) and *N* gene (Right) based on the RSCU values for the viruses belonging to the *Coronaviridae* family. The RSCU values were generated from the CodonW v. 1.4.2 software. Colors represent over-represented and under-represented codons for the genes analyzed. The under-represented genes synonymous codons (RSCU < 0.6) are in green color, Over-represented synonymous codons (RSCU > 1.6) are in Red, the rest are in blue. Abbreviation in figure legends: Porcine hemagglutinating encephalomyelitis virus (PHEV); Mouse hepatitis virus (MHV); Wuhan seafood market pneumonia virus (WSMPV).

RSCU values were calculated to determine the preference of each nucleotide at the wobble position. Figure 1 shows the RSCU plots for the structural genes “*S*” and “*N*” of viruses belonging to the *Coronaviridae* family, in the form of heat maps. It is observed that the most abundantly used codons are different for each virus, indicating that each virus has optimized its codon usage. It was observed that overall, U-ended codons (UUU, CUU, AUU, UCU, ACU and GUU) are over-represented while the G-ended codons (CAG, CCG, GUG, AAG, CGG and GGG) are under-represented. The amino acids Leu and Val are encoded in higher numbers for the different genes analyzed, whereas Trp and His are the least encoded amino acid. However, it is interesting to observe that almost all the viruses have AGA codon encoding for Arginine over-represented for different genes analyzed. 

We further analyzed which codons are either over-represented or under-represented. It is observed that most of the U-ended codons are over-represented whereas a few of the G-ended codons are under-represented in different viruses. RSCU values of the *M* and *E* genes are depicted in Supplementary Figure S1, while Supplementary Figure S2 depicts the RSCU values for the *ORF1a* and *ORF3a* genes for the different viruses analyzed.

### 3.3. Role of Mutational Pressure on the Codon Selection

#### The Effective Number of Codon (ENc) Analysis

**Figure 2:**
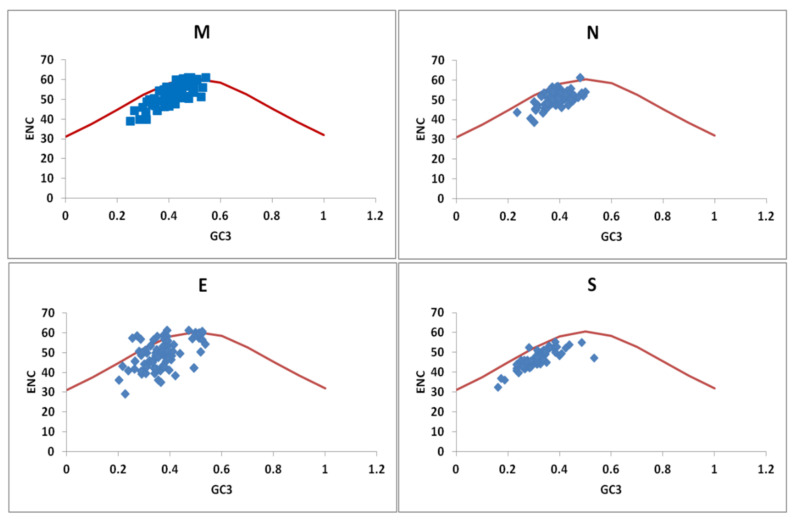
ENc plot for the different structural genes of the *Coronaviridae* family: ENc values for the different genes are plotted against the GC3 values. The expected ENC vs. GC3 plot is depicted as the red-colored curve, whereas the observed ENC vs. GC3 values for the particular virus are plotted as the blue color points. The values for the Enc were derived using the CodonW v. 1.4.2 software.

We further examined the effect of mutational pressure on the extent of variation in synonymous codon usage by plotting GC3 values against ENc values for each set of genes. ENc value on or below the standard curve determines the effect of mutation pressure acting on the gene (Kumar et al., 2016). Figure 2 depicts the GC3 values against the ENc values for the structural genes. It can be seen that ENc values for the *S* and *N* genes lie on the left side of the standard curves below the expected curve. The ENc values for the *M* and the *E* genes also lie below the expected curve, but some fall adjacent. The ENc value indicates the role of mutational pressure, along with other factors in shaping the CUB. The standard deviation of observed ENc values for *S, N, M* and *E* genes are 3.88, 3.74, 5.45 and 7.29 respectively. The mean ENc value of the genes ranges from 46.45 ± 3.88 to 53.33 ± 5.45 (Table 2). It is observed that there is a more significant deviation of the ENc value for the *E* gene (7.29) followed by the *M* gene (5.45) and *S* gene (3.88), indicating that overall the ENc values are not conserved for these genes. 

We further carried out the correlation analysis between the GC3 and GC12, GC and ENc for structural and non-structural genes along with *ORF8* (Table 2). A positive correlation was observed between GC12 and GC for all the structural and non-structural genes. GC12 vs. GC of the *ORF8* gene had a lower correlation: *r* = 0.44, *P* < 1.33 × 10^−7^. GC12 has a non-significant positive correlation with the GC3 nucleotides for the different genes under comparison except for the *ORF1A* and *S* gene, which has a correlation above 0.6 with *p*-value < 0.05.

The correlation between the ENc and the GC12 position was positive, albeit the value of correlation was low (Table 2). An insignificant positive correlation of GC12 vs. GC: *r* = 0.266, *p* = 0.796 (Table 2) was observed. A correlation value of more than 0.5 was observed for the ENc and the third GC codon position. *ORF8* had a non-significant positive correlation with the GCs and the ENc values but demonstrated a strong positive correlation with the GC3 nucleotides.

### 3.4. Role of Natural Selection on Codon Selection

#### 3.4.1. Parity Rule 2 + Analysis

**Figure 3:**
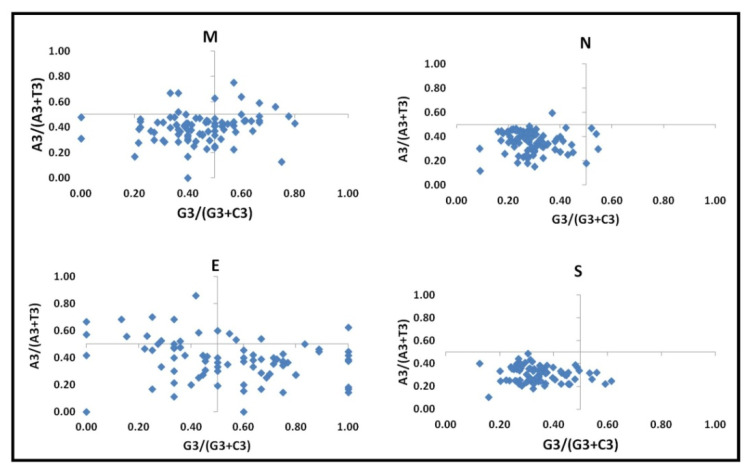
Parity rule-2 bias plot for the different structural genes of the *Coronaviridae* family: The nucleotide frequency at the third position of the four-fold degenerate amino acid was obtained using the MEGA software v. 7.0. A3/(A3 + T3) vs. G3/(G3 + C3) obtained for the different viruses are plotted as a blue point. The center of the ordinate and the abscissa is 0.5, which depicts unbiased usage of the codon.

Parity rule 2 analysis (PR-2 plot) was used to determine the role of natural selection in influencing the codon usage of different viruses in the *Coronaviridae* family (Figure 3). It was observed that the pyrimidine bases were used more than the purine bases for the *N* and *S* genes. *E* and *M* genes have more of the Uracil and Guanine nucleotides. The unequal distribution of the purine and pyrimidine nucleotides for the four-fold degenerate amino acids suggests that factors other than compositional bias may affect the codon usage bias.

#### 3.4.2. Neutrality Plots

**Figure 4:**
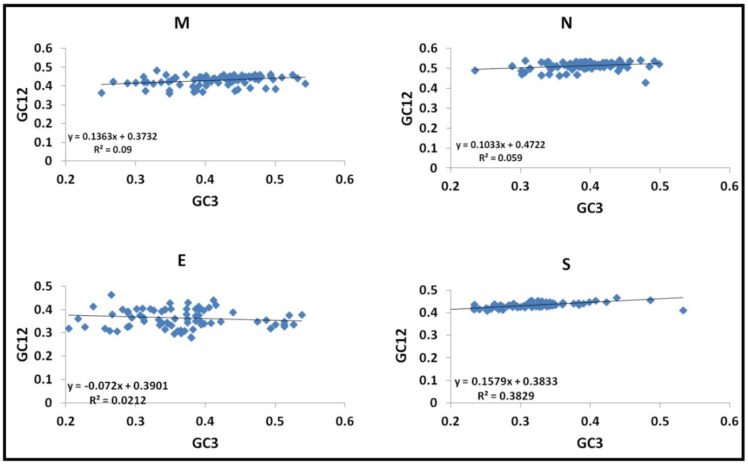
Neutrality plot for the different structural genes of the *Coronaviridae* family: The nucleotide frequency of GC at the first two codon positions vs. GC content at the third codon position is plotted for different genes. The GC12 vs. GC3 values for viruses are depicted as a blue point. The slope of the linear regression through these points denotes the mutational pressure for different genes analyzed.

The GC3-Enc plot demonstrated the role of mutational pressure in shaping the codon usage pattern of the genes, as observed in Figure 4. Further, a neutrality analysis was performed to determine the key factor (natural selection or mutational pressure) determining the shape of codon usage bias (Figure 4). In a neutrality plot, if the slope of the regression line is close, mutational pressure governs the codon usage bias.

The slopes of the regression line calculated for *M, N, S* and *E* genes are found to be 0.136, 0.103, −0.072 and 0.158, respectively. Thus, the slope observed for the genes suggests the role of mainly natural selection being present at the codon positions. The slope of the *E* gene was followed by the *N* gene and indicated a mutational pressure of 7.2% and 10.3%, respectively. The relative neutrality (natural selection) was calculated to be 92.8% and 89.7%, indicating that natural selection plays a dominant role in determining the shape of codon usage bias. Likewise, the *M* gene and *S* gene have a mutational pressure of 13.6% and 15.8%, also indicating the role of natural selection in shaping the codon usage bias.

## 4. Discussion

Genetic degeneracy leads to the usage of different codons for the same amino acid within a gene. The preference of specific codons in organisms leads to uneven use of the codon set and is specific to the organism [27]. Further, the shape of codons usage bias for a gene is governed by evolutionary constraints. The evolutionary constraints structuring the mechanistic details of the codon are the balance between the mutational pressure and natural selection pressure. This work demonstrates the role of evolutionary pressure on the structural genes and accessory genes of the viruses belonging to the *Coronaviridae* family. 

The structural genes of the viruses analyzed from the *Coronaviridae* family were found to possess >50% of pyrimidine nucleotides except for the *N* gene. The genes had >60% of the AU nucleotide except for the *M* and *N* gene that had 57.6% and 52.9% AU, indicating that the family possesses AU rich genomes. The study of mutation pattern between the SARS-CoV-2 and *Bat Coronavirus RaTG13* indicated a strong C > U biased which might be under the influence of the host factors whereby changing the mutational profile [28]. Thus, it is not so surprising that the codons are skewed towards AU (as opposed to GCs) in their genomes (Table 1B and Figure 1). An earlier published study suggests higher AU nucleotides enhance the mutational selection pressure of the gene [29], indicating the role of mutational pressure in the selection of codon usage. A recently published study suggests the role of natural selection in shaping the transmembrane polypeptide that emerged due to top Uracil rich non-genic regions [30], However, the effect of Uracil rich regions in the genic region need to be further looked upon.

Relative synonymous codon usage analysis demonstrated that the abundantly used codons for genes are different for each virus under analysis, which indicated that each virus has a different set of codons that are optimized for their usage. RSCU based analysis led to the identification of a few over-represented and under-represented, U-ended codons and G-ended codons, respectively, for the *Coronaviridae* viruses under study. Differential usage of codons is observed in the structural genes of the *Coronaviridae* family, and it can be proposed that the usage of over-expressed codons might be influenced by the nucleotide composition of the codons. The S protein region has a higher amount of Ser, Thr, and Asn, which is indicative of the presence of glycosylation sites whereas the N-linked glycosylation is reported in the *S* gene of SARS-CoV-2 has been linked to immune evasion and protein folding [31]. Different studies reported the effect of single nucleotide changes in the receptor-binding domain (RBD) region to alter the viral entry in human angiotensin-converting enzyme 2 (ACE2) cells. These mutations can either reduce the serological response, immunogenicity of the virus, or generate an escape mutant virus [32,33,34].

Different methods were used to demonstrate the role and the extent of variation in codon usage bias caused by the mutational pressure and/or natural selection of a gene. ENc has a negative correlation with CUB. Most of the genes studied here belonging to the *Coronaviridae* family have high ENc (close to 50) indicating lesser codon usage bias, suggestive of lesser mutational pressure. A lower ENc value observed for the *ORF1a* (data not shown) and the *S* gene suggests a higher bias in the codon usage of these two genes compared to the other structural genes. The ENc value also indicates that the bias observed in the codon usage is not related to the gene expression [35].

Further, neutrality-based analysis demonstrated that both mutational pressure and natural selection had their role in influencing the codon usage pattern. Earlier work by Zhang et al. demonstrated adaptive evolution of the coronaviruses spike gene, due to positive selection pressure [36]. However, the selection pressures experienced by the different domains in the *S* gene are varied. It was observed that the RBD of the *S* gene has a higher number of positively selected sites in the SARS-CoV-1 [36]. Analysis of the SARS-CoV-2 and the *RaTG13* demonstrated the positive selection to be concentrated in the region that mediates host ACE2receptor binding [37]. Analysis of the different functional proteins by Tang et al. demonstrated positive selection on the spike protein. The comparison carried by the group clustered the other structural genes with the accessory genes and hence were unable to look upon the effect of selection pressure with respect to the other structural genes [38]. As against the previous observation, maximum mutational pressure in the structural genes was observed for the spike gene and least for the *E* gene in this study. This indicated other structural genes have more influence of natural selection as compared to the *S* gene. The other genes analyzed *ORF1a* and *ORF8* had 20% and 3% mutational pressure (data not shown), indicating that natural selection has a minimum of 80% effect on shaping the codon usage of the different genes analyzed. It was observed that the *ORF8* gene was under maximum selection pressure, which is in agreement with the work published by Velazquez-Salinas et al. [39]. They demonstrated that the *ORF8* gene of SARS-CoV-2 had higher dN/dS, and the Leu84Ser, which delineated the strains into the “S” and “L” lineages, indicated a directional selection pressure. The accessory *ORF8* gene plays an important role in the innate immune response influencing viral pathogenicity [39,40]. On the other hand, Hughes and Hughes demonstrated the existence of purifying selection to be prominent in RNA viruses compared to DNA viruses [41].

## 5. Conclusions

Overall, the study demonstrated that the codon usages in the structural genes of viruses from the *Coronaviridae* family are biased. The major factor that shapes the codon usage is natural selection. The *ORF1a* gene and *S* gene were noted to have a combined effect from both the mutational as well as natural selection, while natural selection contributed majorly to a majority of the structural genes in shaping the codon usage.

## Figures and Tables

**Table 1:** Mean and standard deviation of nucleotides and dinucleotides in different gene sets along with standard deviation.

**(A) Mean Occurrence of Individual Nucleotide along with Standard Deviation**								
**Gene**	**T(U)**	**C**	**A**	**G**				
***E***	37.7 ± 3.5	17.9 ± 3.2	25.4 ± 2.7	19 ± 1.8				
***M***	32.3 ± 2.5	20.4 ± 2.6	25.3 ± 1.8	21.8 ± 1.3				
***N***	23.1 ± 2.4	23.9 ± 2.3	29.8 ± 1.9	23.0 ± 1.7				
***S***	33.6 ± 2.3	19.3 ± 2.4	27.2 ± 2.0	19.6 ± 1.4				
***ORF1A***	33.5 ± 2.5	17.5 ± 2.5	26.8 ± 1.9	22.0 ± 1.4				
***ORF1B***	32.8 ± 2.0	17.6 ± 2.1	28.2 ± 1.4	21.2 ± 1.1				
***ORF3A***	34.7 ± 3.3	19.9 ± 2.6	25.9 ± 2.5	19.3 ± 1.6				
***ORF8***	33.2 ± 1.7	18.9 ± 1.3	29.6 ± 1.4	18.2 ± 1.2				
**(B) Mean Occurrence of Dinucleotide at Different Codon Positions along with Standard Deviation**								
**Nucleotide (%)**	**GC**	**AU**	**GC1**	**GC2**	**GC3**	**AU1**	**AU2**	**AU3**
***E***	36.9 ± 3.5	63.0 ± 3.5	45.2 ± 4.1	30.9 ± 4.6	34.5 ± 5.4	54.7 ± 4.1	69.0 ± 4.6	65.4 ± 5.4
***M***	42.3 ± 3.1	57.6 ± 3.1	45.1 ± 4.3	40.6 ± 2.2	41.2 ± 6.1	54.8 ± 4.3	59.3 ± 2.2	58.7 ± 6.1
***N***	47.0 ± 2.4	52.9 ± 2.4	54.4 ± 2.3	47.9 ± 3.0	38.5 ± 4.9	45.5 ± 2.3	52.0 ± 3.0	61.4 ± 4.9
***S***	39.0 ± 2.7	60.9 ± 2.7	45.7 ± 2.1	40.7 ± 1.3	30.6 ± 5.8	54.2 ± 2.1	59.2 ± 1.3	69.3 ± 5.8
***ORF1A***	39.6 ± 2.8	60.3 ± 2.8	48.7 ± 2.2	38.0 ± 1.7	31.9 ± 5.8	51.2 ± 2.2	61.9 ± 1.7	68.0 ± 5.8
***ORF1B***	38.9 ± 2.2	61.0 ± 2.2	47.0 ± 1.5	37.4 ± 0.8	32.2 ± 4.7	52.9 ± 1.5	62.5 ± 0.8	67.7 ± 4.7
***ORF3A***	39.3 ± 2.7	60.6 ± 2.7	46.9 ± 3.8	36.5 ± 5.2	34.3 ± 6.1	53.0 ± 3.8	63.4 ± 5.2	65.6 ± 6.1
***ORF8***	37.1 ± 2.0	62.8 ± 2.0	49.8 ± 3.3	34.2 ± 2.2	27.2 ± 4.4	50.1 ± 3.3	65.7 ± 2.2	72.7 ± 4.4

**Table 2:** Mean ENc and Correlation between various parameters analyzed for a different set of genes in the *Coronaviridae* family.

Genes	Mean ENC	Correlation of GC12 with GC3	Correlation of GC12 with GC	Correlation of GC12 with ENc	Correlation of ENc with GC3
***E***	49.0	0.15 *p*-value (1.68 × 10^−33^)	0.66 *p*-value (0.011)	0.014 *p*-value (1.07 × 10^−65^)	0.53 *p*-value (0.058)
***M***	53.3	0.3 *p*-value (8.73 × 10^−34^)	0.78 *p*-value (4.75 × 10^−7^)	0.27 *p*-value (4.67 × 10^−22^)	0.78 *p*-value (0.208)
***N***	50.7	0.24 *p*-value (1.55 × 10^−45^)	0.75 *p*-value (4.03 × 10^−10^)	0.21 *p*-value (1.56 × 10^−28^)	0.54 *p*-value (1.37 × 10^−20^)
***S***	46.5	0.62 *p*-value (4.58 × 10^−72^)	0.82 *p*-value (2.59 × 10^−33^)	0.60 *p*-value (3.08 × 10^−37^)	0.78 *p*-value (2.08 × 10^−37^)
***ORF1A***	47.0	0.70 *p*-value (1.07× 10^−65^)	0.87 *p*-value (1.57 × 10^−31^)	0.43 *p*-value (4.39 × 10^−32^)	0.66 *p*-value (3.48 × 10^−29^)
***ORF8***	48.1	0.11 *p*-value (0.062)	0.44 *p*-value (1.33 × 10^−7^)	0.33 *p*-value (1.61 × 10^−25^)	0.89 *p*-value (0.010)

## Supplementary Materials

The following are available online at https://www.mdpi.com/1999-4915/13/1/3/s1, Figure S1. Heat map of the *M* and *E* genes based on relative synonymous codon usage (RSCU) values: A heat map plot of *M* (Left) and *E* gene (Right) based on the RSCU values for the viruses belonging to the *Coronaviridae* family. The RSCU values were generated from the CodonW v1.4.2 software. Colors represent over-represented and under-represented codons for the genes analyzed. The under-represented genes (RSCU > 0.6) are in green color, over-represented (RSCU < 1.6) are in red, and the rest are in blue, Figure S2. Heat map of the *ORF1a* and *ORF3a* genes based on relative synonymous codon usage (RSCU) values: A heat map plot of *ORF1a* (Left) and *ORF3a* gene (Right) based on the RSCU values for the viruses belonging to *Coronaviridae* family. The RSCU values were generated from the CodonW v1.4.2 software. Colors represent over-represented and under-represented codons for the genes analyzed. The under-represented genes (RSCU > 0.6) are in green color; over-represented (RSCU < 1.6) are in red, and rest are in blue, Table S1. Accession number of the viruses from the *Coronaviridae* family analyzed in this study.
