# Supplementary material for: Natural Selection Plays an Important Role in Shaping the Codon Usage of Structural Genes of the Viruses Belonging to the Coronaviridae Family
Source: Viruses. 2020 Dec 22;13(1):3. doi: 10.3390/v13010003 (PMC7821998; doi:10.3390/v13010003)
Supplement: Supplementary file 1 [file viruses-13-00003-s001.pdf]

Supplementary Table S1. Accession number of the viruses from the *Coronaviridae* family analyzed in this study.

Accession no.	Name	Genus	Sub-Genus
MN975262.1	SARS-CoV- 2 isolate 2019-nCoV_HKU-SZ-005b_2020	β-Cov	Sarbecovirus
NC_045512.2	WSMPV isolate Wuhan-Hu-1	β-Cov	Sarbecovirus
NC_039208.1	Porcine coronavirus HKU15 strain HKU15-155	δ-Cov	Buldecovirus
NC_039207.1	Betacoronavirus Erinaceus/VMC/DEU/2012 isolate ErinaceusCoV/2012-174/GER/2012	β-Cov	Merbecovirus
NC_038861.1	Transmissible gastroenteritis virus	α-Cov	Tegacovirus
NC_034972.1	Coronavirus AcCoV-JC34	α-Cov	unclassified
NC_032730.1	Lucheng Rn rat coronavirus isolate Lucheng-19	α-Cov	Luchacovirus
NC_032107.1	NL63-related bat coronavirus strain BtKYNL63-9a	α-Cov	Setracovirus
NC_038294.1	Betacoronavirus England 1	β-Cov	Merbecovirus
NC_035191.1	WenchengSm shrew coronavirus isolate Xingguo-101	α-Cov	unclassified
NC_030886.1	Rousettus bat coronavirus isolate GCCDC1 356	β-Cov	Nobecovirus
NC_030292.1	Ferret coronavirus isolate FRCoV-NL-2010	α-Cov	Minacovirus
NC_028833.1	BtNv-AlphaCoV/SC2013	α-Cov	Nyctacovirus
NC_028824.1	BtRf-AlphaCoV/YN2012	α-Cov	unclassified
NC_028814.1	BtRf-AlphaCoV/HuB2013	α-Cov	Decacovirus
NC_028811.1	BtMr-AlphaCoV/SAX2011	α-Cov	Myotacovirus
NC_028806.1	Swine enteric coronavirus strain Italy/213306/2009	α-Cov	Tegacovirus
NC_028752.1	Camel alphacoronavirus isolate camel/Riyadh/Ry141/2015	α-Cov	Duvinacovirus
NC_026011.1	Betacoronavirus HKU24 strain HKU24-R05005I	β-Cov	Embecovirus
NC_025217.1	Bat Hp-betacoronavirus/Zhejiang2013	β-Cov	Hibecovirus
NC_023760.1	Mink coronavirus strain WD1127	α-Cov	Minacovirus
NC_022103.1	Bat coronavirus CDPHE15/USA/2006	α-Cov	Colacovirus
NC_019843.3	Middle East respiratory syndrome coronavirus	β-Cov	Merbecovirus
NC_018871.1	Rousettus bat coronavirus HKU10	α-Cov	Decacovirus
NC_017083.1	Rabbit coronavirus HKU14	β-Cov	unclassified
NC_016996.1	Common-moorhen coronavirus HKU21	δ-Cov	Moordecovirus
NC_016995.1	Wigeon coronavirus HKU20	δ-Cov	Andecovirus
NC_016994.1	Night-heron coronavirus HKU19	δ-Cov	Herdecovirus
NC_016993.1	Magpie-robin coronavirus HKU18	δ-Cov	unclassified
NC_016992.1	Sparrow coronavirus HKU17	δ-Cov	unclassified
NC_016991.1	White-eye coronavirus HKU16	δ-Cov	Buldecovirus
NC_012936.1	Rat coronavirus Parker	β-Cov	Embecovirus
NC_011550.1	Munia coronavirus HKU13-3514	δ-Cov	Buldecovirus
NC_011549.1	Thrush coronavirus HKU12-600	δ-Cov	unclassified
NC_011547.1	Bulbul coronavirus HKU11-934	δ-Cov	Igacovirus
NC_010800.1	Turkey coronavirus	γ-Cov	Turkey coronavirus
NC_010646.1	Beluga Whale coronavirus SW1	γ-Cov	Cegacovirus
NC_010438.1	Bat coronavirus HKU8	α-Cov	Minunacovirus
NC_010437.1	Bat coronavirus 1A	α-Cov	unclassified
NC_009988.1	Bat coronavirus HKU2	α-Cov	Rhinacovirus
NC_009657.1	Scotophilus bat coronavirus 512	α-Cov	Pedacovirus
NC_009021.1	Bat coronavirus HKU9-1	β-Cov	Nobecovirus
NC_009020.1	Bat coronavirus HKU5-1	β-Cov	Merbecovirus
NC_009019.1	Bat coronavirus HKU4-1	β-Cov	Merbecovirus
NC_006577.2	Human coronavirus HKU1<br>Human coronavirus HKU1	β-Cov	Embecovirus
NC_006213.1	Human coronavirus OC43 strain ATCC VR-759	β-Cov	Embecovirus

NC_005831.2	Human Coronavirus NL63	α-Cov	Setracovirus
NC_003436.1	Porcine epidemic diarrhea virus	α-Cov	Pedacovirus
NC_003045.1	Bovine coronavirus	β-Cov	Embecovirus
NC_002645.1	Human coronavirus 229E	α-Cov	Duvinacovirus
NC_001451.1	Avian infectious bronchitis virus	γ-Cov	Igacovirus
AC000192.1	Murine hepatitis virus strain JHM	β-Cov	Embecovirus
NC_002306.3	Feline infectious peritonitis virus	α-Cov	Tegacovirus
NC_001846.1	Mouse hepatitis virus strain MHV-A59 C12 mutant	β-Cov	Embecovirus
MN988713.1	SARS-CoV-2 isolate 2019-nCoV/USA-IL1/2020	β-Cov	Sarbecovirus
MN985325.1	SARS-CoV-2 isolate 2019-nCoV/USA-WA1/2020	β-Cov	Sarbecovirus
MN938384.1	SARS-CoV-2 isolate 2019-nCoV_HKU-SZ-005b_2020	β-Cov	Sarbecovirus
KC869678.4	SARS-CoV-2 isolate 2019-nCoV_HKU-SZ-002a_2020, complete genome.	β-Cov	Sarbecovirus
DQ022305.2	SARS-CoV-2 isolate Wuhan-Hu-1	β-Cov	Sarbecovirus
DQ071615.1	Coronavirus Neoromicia/PML-PHE1/RSA/2011	β-Cov	Merbecovirus
DQ412042.1	Bat SARS coronavirus HKU3-1	β-Cov	Sarbecovirus
DQ412043.1	Bat SARS coronavirus Rm1	β-Cov	Sarbecovirus
EF065513.1	Bat coronavirus HKU9-1	β-Cov	Nobecovirus
FJ588686.1	Bat SARS CoV Rs672/2006	β-Cov	Sarbecovirus
HQ166910.1	Zaria bat coronavirus strain ZBCoV	β-Cov	unclassified
HQ728482.1	Eidolon bat coronavirus/Kenya/KY24/2006	β-Cov	unclassified
HQ728483.1	Rousettus bat coronavirus/Kenya/KY06/2006	β-Cov	unclassified
JX993987.1	Bat coronavirus Rp/Shaanxi2011	β-Cov	unclassified
JX993988.1	Bat coronavirus Cp/Yunnan2011	β-Cov	unclassified
KC243390.1	Bat coronavirus BtCoV/8-724/Pip_pyg/ROU/2009	β-Cov	unclassified
KC881005.1	Bat SARS-like coronavirus RsSHC014	β-Cov	unclassified
KC881006.1	Bat SARS-like coronavirus Rs3367	β-Cov	Sarbecovirus
KF367457.1	Bat SARS-like coronavirus WIV1	β-Cov	Sarbecovirus
KJ473821.1	BtVs-BetaCoV/SC2013	β-Cov	unclassified
NC_014470.1	Bat coronavirus BM48-31/BGR/2008	unclassified	unclassified
KP886808.1	Bat SARS-like coronavirus YNLF_31C	β-Cov	Sarbecovirus
MG693170.1	Bat coronavirus isolate CMR66	unclassified	unclassified
MG772933.1	Bat SARS-like coronavirus isolate bat-SL-CoVZC45	β-Cov	Sarbecovirus
MG772934.1	Bat SARS-like coronavirus isolate bat-SL-CoVZXC21	β-Cov	Sarbecovirus
MK211379.1	Coronavirus BtRt-BetaCoV/GX2018	β-Cov	unclassified
MK492263.1	Bat coronavirus strain BtCoV92	unclassified	unclassified
NC_034440.1	Bat coronavirus isolate PREDICT/PDF-2180	unclassified	unclassified
JX869059.2	Human betacoronavirus 2c EMC/2012	β-Cov	Merbecovirus
NC_005147.1	Human coronavirus OC43	β-Cov	unclassified
KJ477102.1	Middle East respiratory syndrome coronavirus	β-Cov	unclassified
KJ713299.1	Middle East respiratory syndrome coronavirus isolate KSA-CAMEL-376	β-Cov	unclassified
NC_010327.1	Equine coronavirus	β-Cov	unclassified
NC_007732.1	Porcine hemagglutinating encephalomyelitis virus	β-Cov	unclassified
KF294357.1	Longquan Aa mouse coronavirus isolate Longquan-343	β-Cov	unclassified
KF294370.1	LongquanRl rat coronavirus isolate Longquan-189	β-Cov	unclassified
AY304486.1	SARS coronavirus SZ3	β-Cov	Sarbecovirus

Legend for the Supplementary Figures

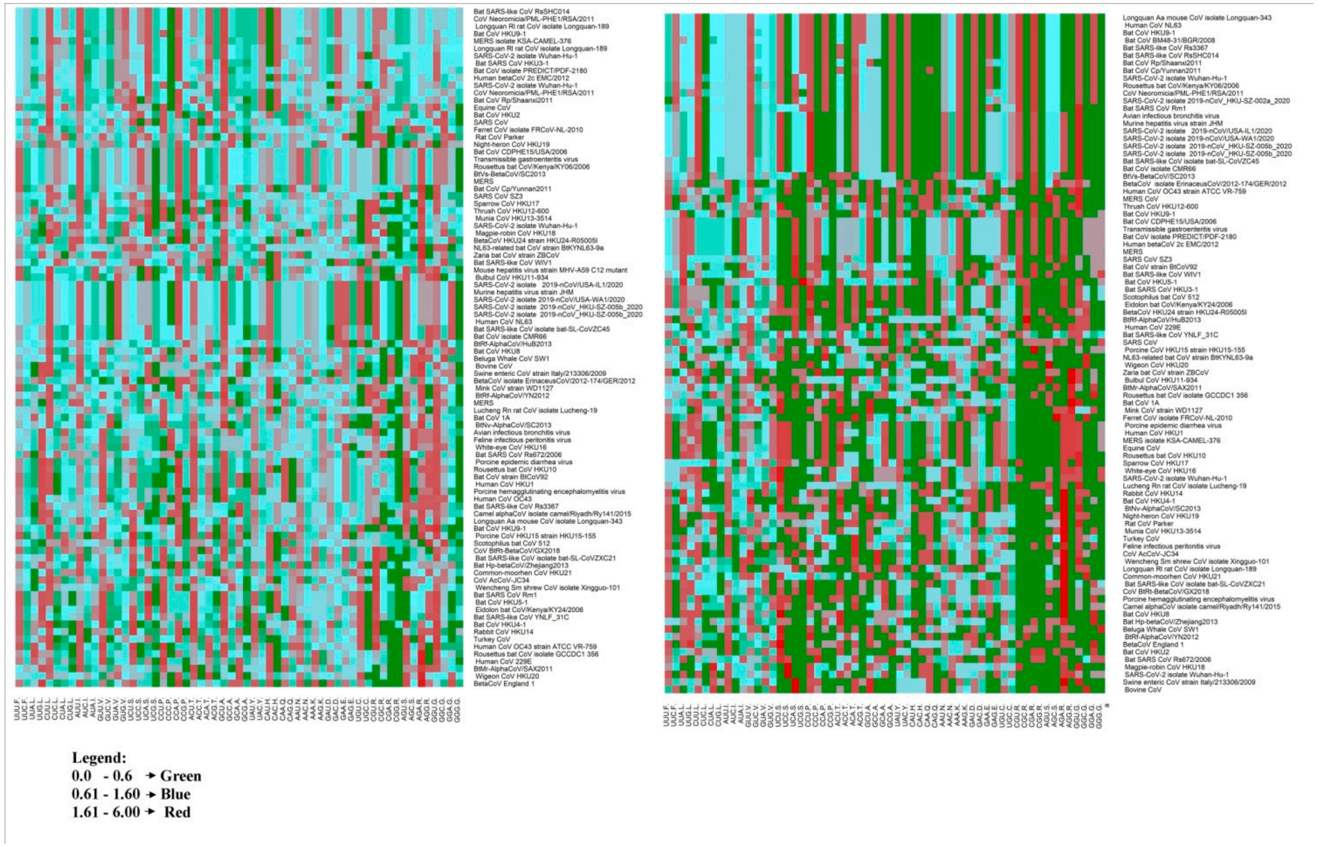

Supplementary Figure S1. Heat map of the *M* and *E* genes based on relative synonymous codon usage (RSCU) values: A heat map plot of *M* (Left) and *E* gene (Right) based on the RSCU values for the viruses belonging to the *Coronaviridae* family. The RSCU values were generated from the CodonW v1.4.2 software. Colors represent over-represented and under-represented codons for the genes analyzed. The under-represented genes (RSCU>0.6) are in green color, over-represented (RSCU<1.6) are in red, and the rest are in blue.

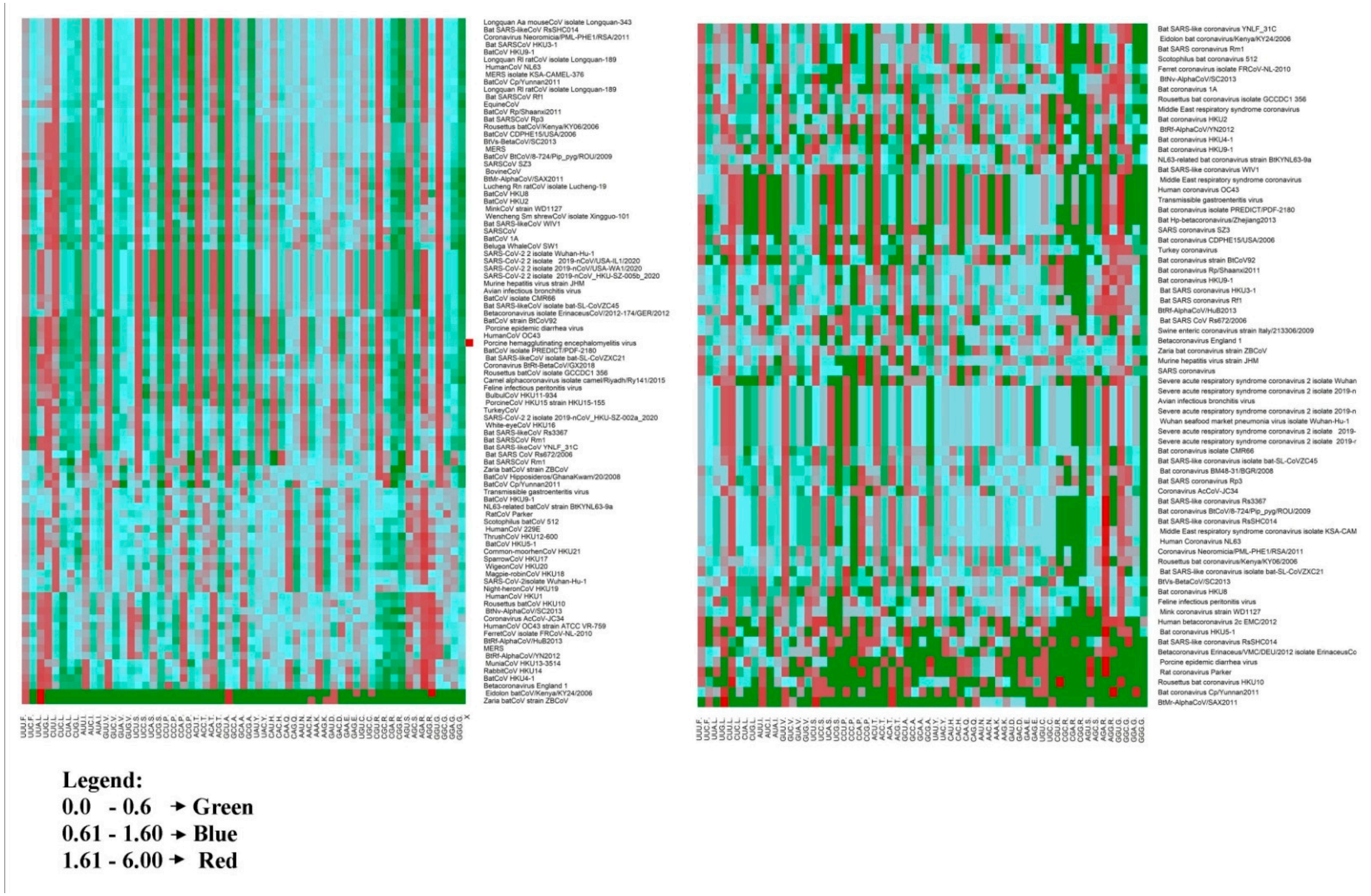

Supplementary Figure S2. Heat map of the *ORF1a* and *ORF3a* genes based on relative synonymous codon usage (RSCU) values: A heat map plot of *ORF1a* (Left) and *ORF3a* gene (Right) based on the RSCU values for the viruses belonging to *Coronaviridae* family. The RSCU values were generated from the CodonW v1.4.2 software. Colors represent over-represented and under-represented codons for the genes analyzed. The under-represented genes (RSCU>0.6) are in green color;over-represented (RSCU<1.6) are in red, and rest are in blue.
